# Hybrid Junction-Enabled Biomimetic Human Eye Structure for Large Dynamic Range Vision Sensor

**DOI:** 10.3390/nano16090498

**Published:** 2026-04-22

**Authors:** Daqi Chen, Yueheng Lu, Zhenye Zhan, Yuanfan Han, Zhendong Weng, Jian Chen, Qiulan Chen, Yang Zhou, Weiguang Xie

**Affiliations:** 1 Siyuan Laboratory, Guangdong Provincial Engineering Technology Research Center of Vacuum Coating Technologies and New Energy Materials, Department of Physics, Jinan University, Guangzhou 510632, China; 2 Instrumental Analysis & Research Center, Sun Yat-sen University, Guangzhou 510275, China; 3 Department of Medical Devices, Guangdong Food and Drug Vocational College, Guangzhou 510520, China; 4 Guangdong Provincial Key Laboratory of Optical Fiber Sensing and Communications, Jinan University, Guangzhou 510632, China

**Keywords:** WSe_2_, VO_2_, heterojunction, dynamic range, vision sensor, photodiode, van der Waals contacts

## Abstract

The responsive light intensity dynamic range (DR) of the human eye far exceeds that of existing visual systems, and the development of a biomimetic retinal detecting unit is currently an important challenge in the field of machine vision. Here, a two-terminal Au-contacted VO_2_/WSe_2_ heterojunction photodetector with the same adaptive DR as retinal cells is developed. It is revealed that the VO_2_/WSe_2_ heterojunction part-mimics the cone cell for strong light detection with photoresponsivity (R) of 320 mA W^−1^ and the Au/WSe_2_ Schottky contact part-mimics the rod cell for weak light detection with an R of 217 A W^−1^ and noise equivalent power (NEP) as low as 248.2 fW/√Hz. The dual-mode photodetector shows a fast response speed of less than 39.28 μs. Image fusion by the cone mode and rod mode shows enhanced recognition. These results demonstrate that contact engineering enables a photodetector with the functionality of both rod and cone cells, and the resulting visual imaging system can achieve performance comparable to that of the human eye in certain operating conditions.

## 1. Introduction

Machine vision systems used in applications such as unmanned aerial vehicles, smart cars and intelligent robots are required to complete tasks such as object detection and recognition in a broad light intensity range [1,2,3,4,5]. Traditional complementary metal oxide semiconductor imagers sense light intensity with a small dynamic range (DR) of 60–70 dB in natural light, with a broad DR of 280 dB [6,7,8]. In illumination conditions with a large DR, the traditional imager captures bright light information at the expense of weak light information, and vice versa [9,10,11]. In contrast, the visual cell of human eyes is composed of a strong light-sensitive cone cell and a weak light-sensitive rod cell. Light intensity adaptation and image fusion by both cone and rod cells expand the detection and recognition of DR to 140 dB [12,13,14]. The realization of a basic unit with biomimetic hybrid sensing and image fusion functionality in human eyes is a challenging topic [15,16,17,18].

In recent years, several bio-inspired device structures and mechanisms have been proposed to mimic the retinal cells [11,19,20]. For instance, Zhong et al. proposed an ion/electron-tuned CuInP_2_S_6_ visual sensor, which achieves regulation through the coupling of ion migration and electron photogeneration processes. It exhibits a dependency on current and time, as well as light intensity, and can independently achieve visual adaptation and target extraction in different light intensity scenarios without relying on post-processing equipment [12]. Liu et al. proposed a self-powered wide-spectrum bionic visual sensor based on multi-layer γ-InSe, which achieved dynamic adaptive behavior in response to continuous and constant light stimulation through the pyroelectric effect [13]. Although these outstanding advances have brought new ideas to the development of novel machine vision devices and laid the foundation for subsequent research and development, they did not show the dynamic range of the devices, which is one of the key parameters in visual sensors. To explore the specific dynamic ranges, Liao et al. introduced charge trapping states in a molybdenum disulfide phototransistor and switched between negative and positive gate bias to mimic the rod and cone behavior. The device successfully realizes the visual adaptation behavior of human eyes with a perception DR of 199 dB [9]. Zhou et al. proposed switching between photoconductive (rod) and photodiode (cone) mode through band alignment tuning by both gate and source/drain bias in a silicon/graphene phototransistor. The device is not self-adaptive but combines both modes to produce DR of 170 dB [8]. To further simplify the device structure, two-terminal photoconductive/diode devices with structures more analogous to the human eye have also been developed. Ran et al. proposed a biomimetic dual-mode visual sensor based on Cd (S, Se) nanowires, in which sulfur vacancy-induced trap states enable spontaneous switching between transient and persistent photoconductive behaviors. The device achieved a dynamic range of 198 dB, demonstrating that two-terminal devices can mimic the fundamental operating principles of the human eye [6]. It can be deemed that tentative investigation has been done to mimic the functions of the human eye in different ways; however, the device structure, mechanism, performance, control methods, etc., still require intense exploration to bring the visual sensor into use.

In this work, we demonstrate a simple two-terminal Au-contacted VO_2_/WSe_2_ heterojunction device to mimic the structure of the retinal cell. In the device, the photoresponsive area switches between the VO_2_/WSe_2_ heterojunction and the WSe_2_/Au Schottky junction with the VO_2_/Au as a non-responsive Ohmic contact area. The heterojunction and Schottky junction mimic the cone cell and the rod cell respectively, extending the sensing range to 146.66 dB with a response speed of 27.77 μs. The low-bias rod mode and zero-bias cone mode offer the advantage of low-power operation for machine vision.

## 2. Experimental Section

Preparation of materials: VO_2_ crystals were prepared by chemical vapor deposition (CVD). Vanadium pentoxide (V_2_O_5_) powder was used as the reaction source. A total of 0.07 g of V_2_O_5_ powder was weighed and placed in a circular crucible, spread evenly and put into a tube furnace. The growth temperature was 850 °C and the holding time was 150 min [21,22]. Finally, the grown VO_2_ was subjected to a 4 h annealing process at 400 degrees Celsius in a nitrogen atmosphere to further enhance the crystal quality [23]. The final VO_2_ optical microscopic images and XRD are shown in Figure S1. The WSe_2_ crystals were purchased from HQ Company, and the nanosheets were obtained through mechanical exfoliation and dry transfer methods.

Construction of the device: The specific process of device fabrication is shown in Figure S2. Firstly, the grown VO_2_ crystals were transferred onto the SiO_2_ (300 nm)/Si substrate using polydimethylsiloxane (PDMS). Then, thinner WSe_2_ nanosheets were obtained by mechanical exfoliation and precisely stacked on VO_2_ crystals. Subsequently, a mask of appropriate size was used for masking, and finally, Au electrodes were deposited by thermal evaporation.

Characterization and testing: The optical microscope (LABIV-6H, IVTEST, Foshan, China) was used to characterize the surface morphology. Raman and PL spectra were measured by confocal Raman microscopy (Alpha300R, RENISHAW, Gloucestershire, UK) using a 532 nm laser as the excitation source. The photoelectric response characteristics of the device were characterized using a semiconductor parameter analyzer with a semiconductor laser (FS-PRO, PRIMARIUS, Shanghai, China). The photoresponse characteristics of the device were measured using a 405 nm semiconductor laser (LE-LS-VIS, Leo-photoelectric, Shenzhen, China), and the corresponding light intensity was calibrated with an optical power meter (GCI080302, Daheng Optics, Nanjing, China). AFM (Ntegra Prima, NT-MDT, Moscow, Russia) and KPFM modes were integrated for the analysis of morphology and surface potential. All the above measurements were completed at room temperature and in an ambient environment.

## 3. Results and Discussion

### 3.1. Design Principles and Characterization

**Figure 1:**
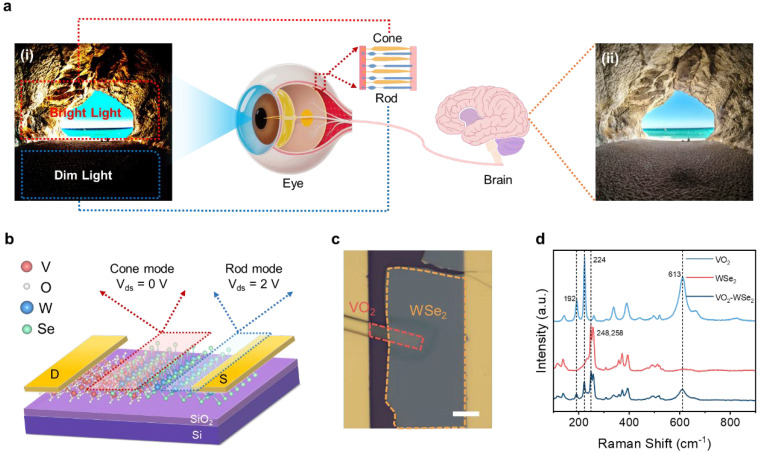
Device design principles and characterization: (**a**) Schematic diagram of the human visual mechanism. (**i**) is an optical image of the cave, conceptually representing the image captured by the human eye and highlighting the light perception characteristics of rod cells and cone cells. (**ii**) is the schematic diagram of the image when the human brain fuses the information captured by rod cells and cone cells. (**b**) Schematic diagram of the structure of Au-contacted VO_2_/WSe_2_ heterojunction photodetector. The 0 V bias simulates the cone cells of the human eye, and the 2 V bias simulates the rod cells of the human eye. (**c**) Optical image of the VO_2_/WSe_2_ heterojunction photodetector. Scale bar, 9 μm. (**d**) Raman spectra at different positions of the heterojunction.

Figure 1a schematically shows the imaging process of human eyes. Both the rod cells and cone cells capture the natural scene with great light intensity difference, and the visual information is input into the cerebral cortex, eventually generating an image with adaptive contrast for the recognition of objects in both dim and strong illumination conditions [14,24,25,26]. Inspired by the dynamic tuning mechanism of cone cells and rod cells in the human retina, a heterojunction photodetector with both bright and dim light-sensitive structures to simulate the adaptive fusion in the human retina is designed, as schematically shown in Figure 1b. The device is composed of a VO_2_ belt and WSe_2_ nanosheet stacked through van der Waals interaction, and Au is used as a contact electrode. At 0 V bias voltage, the VO_2_/WSe_2_ heterojunction region outlined by a red rectangular dotted line behaves as a cone cell, while at 2 V bias voltage, the detector switches to rod cell mode, and the active region changes to the WSe_2_/Au Schottky interface outlined by a blue rectangular dotted line.

Details of the preparation of VO_2_/WSe_2_ and fabrication of the device are shown in Figures S1 and S2. The optical image of the as-prepared VO_2_/WSe_2_ heterojunction photodetector is shown in Figure 1c. The morphology of the heterojunction was analyzed using an atomic force microscope as shown in Figure S3a. The thicknesses of VO_2_ and WSe_2_ are 590 nm and 54 nm, respectively. The Raman spectra of VO_2_ in Figure 1d show typical peaks at 192 cm^−1^ and 224 cm^−1^, corresponding to the V-V key vibration of M1 phase VO_2_, while the peak of 613 cm^−1^ corresponds to the vibration of the V-O key [27,28,29]. The Raman peak of WSe_2_ at 248 cm^−1^ corresponds to the Se-Se key in-plane vibration (E_2g_) and the Raman peak at 258 cm^−1^ corresponds to the W-Se key out-of-plane vibration (A_1g_), identifying the good quality of transferred WSe_2_ [30,31,32]. The heterojunction region exhibited all the Raman peaks of the two materials, which indicates that our heterojunction has been well constructed. Figure S3b shows the exciton emission peak at 774 nm and the indirect bandgap emission peak at 910 nm in WSe_2_ [33,34,35]. The PL peak in the heterojunction region is weaker than that in WSe_2_, which indicates that there is an interlayer coupling of excitons in the heterojunction region [34,36,37]. The results of both Raman and PL spectra indicate that the material quality of the device we constructed is excellent.

### 3.2. Device Performance

**Figure 2:**
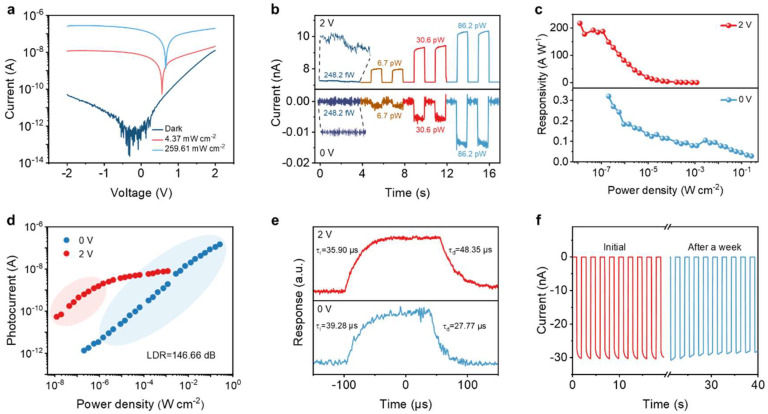
Dual-mode photoresponse performance: (**a**) current–voltage curves of VO_2_/WSe_2_ van der Waals heterojunction photodetectors under different optical powers. (**b**) Current–time curves of heterojunction under 2 V and 0 V bias voltages. (**c**) The variations in the responsivity of heterojunction with the optical power density under the bias voltages of 2 V and 0 V. (**d**) Current–optical power density curves of heterojunction under 2 V and 0 V bias voltages. (**e**) The response speeds of heterojunction at 2 V and 0 V bias voltages. (**f**) The current–time curve of heterojunction after being placed in the air for a week.

We used a semiconductor laser with a wavelength of 405 nm as the light source to test the photoelectric response performance of the device. The intensity of the light was calibrated using a light power meter. The optoelectrical characteristics of the as-prepared VO_2_/WSe_2_ heterojunction photodetector are shown in Figure 2 and Figure S5. The I–V characteristic curves of the device under different light intensities are shown in Figure 2a. The rectification ratio of the device in the dark state exceeds 10^2^, and the maximum light on/off ratio exceeds 10^3^. These results indicate that the VO_2_/WSe_2_ heterojunction exhibits the characteristics of a photodiode. Typical photosensitive performances at V_ds_ = 2 V and V_ds_ = 0 V are compared as the voltages are at both sides of the voltage of minimum photocurrent in Figure 2a. First, the sensitivity of a photodetector is characterized by the noise equivalent power (NEP), which is defined as the incident light power required when the signal-to-noise ratio (SNR) is 1 [38,39]. NEP can be determined by adjusting the current–time curves under different light intensity modulations [40,41]. As shown in Figure 2b, the NEP of the device at V_ds_ = 2 V is 248.2 fW/√Hz, which is much better than 6.7 pW/√Hz at V_ds_ = 0 V. This indicates that the detector can detect even weaker light intensities at 2 V. This phenomenon can be explained using photoconductive gain, with $G=\frac{\tau_{n}}{\tau_{t}}$ [42,43]. $\tau_{n}$ and $\tau_{t}$ represent the lifetime and transit time of the carriers respectively. When $\tau_{n}>\tau_{t}$, each photon absorbed by the detector enables multiple electrons to continue passing through the two electrodes. When the applied voltage is V, the transit time $\tau_{t}=\frac{l^{2}}{\mu_{n}*V}$, where $\mu_{n}$ is the electron mobility, l is the distance between the electrodes, and $G=\frac{\tau_{n}*\mu_{n}*V}{l^{2}}$. Therefore, adding a bias voltage will be beneficial for detecting even weaker light signals. Figure 2c shows that the maximum photoresponsivity is 217 A W^−1^ at 2 V, about 700 times that at 0 V. Second, the photoresponsivity at 2 V decreases rapidly with increasing light intensity. The intensity-dependent photoresponse in Figure S5a shows that the device operating at 2 V is less sensitive when the illumination intensity is higher than 8.85 μW cm^−2^. As a consequence, R decreases to 20.2 A W^−1^ in Figure 2c, which limits its DR to 41.82 dB as shown in Figure S5c. In contrast, the device operating at 0 V effectively responses to strong illumination up to 259.6 mW cm^−2^ in Figure S5b, enabling a larger DR of 122.03 dB as shown in Figure S5d. Compared to the human eye, it can be seen that the as-prepared photodetector operating at 2 V is more sensitive to a weak light signal while less responsive to a strong one, enabling the effective function of the retinal rod cell (rod mode). When operating at 0 V, the broader photoresponse to strong light signals enables the simulation of the retinal cone cell (cone mode). As shown in Figure 2d, the combined DR of the photodetector is 146.66 dB.

As another crucial parameter for evaluating the performance of photodetectors, the response time includes the rise time (t_r_) and the fall time (t_f_). The rise time is defined as the time to rise from 10% to 90% of the photocurrent, and the fall time is defined as the time to fall from 90% to 10% of the photocurrent [44,45]. Figure 2e shows that the rise time and fall time at 0 V are 39.28 μs and 27.77 μs respectively, while the rise/fall time at 2 V are 35.9 μs and 48.35 μs. Such response speed is at a relatively high level. The specific detectivity at 2 V is approximately $1.1\times 10^{9}$ Jones, and the specific detectivity at 0 V voltage is approximately $1.08\times 10^{7}$ Jones. Furthermore, the device shows good ambient stability. After being stored at room temperature in an air environment for a week, the device still maintained a stable, rapid and repeatable photoresponse as shown in Figure 2f. Table S1 shows that our device shows a relatively high level among two-dimensional material photodetectors.

Finally, we compared the VO_2_/WSe_2_ visual sensor with recently reported bio-inspired human-eye visual sensors, as summarized in Table 1. In terms of device architecture, field-effect transistor structures were the earliest biomimetic human-eye configurations to be explored. More recently, however, simpler two-terminal photoconductive/diode devices, whose structures are more analogous to that of the human eye, have emerged. Regarding operating mechanisms, a defect charge-mediated self-adaptation strategy can deliver a larger dynamic range (DR), but usually at the expense of a substantial reduction in response speed and adaption speed to more than one second. In contrast, the switching between devices with different response behaviors generally yields a relatively smaller DR, while enabling response and adaption speed to be achieved in less than a millisecond. Both our device and the recently reported Cd (S, Se) visual sensor [6] demonstrate that two-terminal device architectures can mimic the fundamental operating principles of the human eye and achieve a DR comparable to that of the human eye in different ways. Moreover, our device further shows that a single heterojunction photodiode is able to mimic the rod- and cone-like structure by simply switching the bias, highlighting the significant potential for device design in the development of bio-inspired visual sensors.

### 3.3. Photoresponse Mechanism

**Figure 3:**
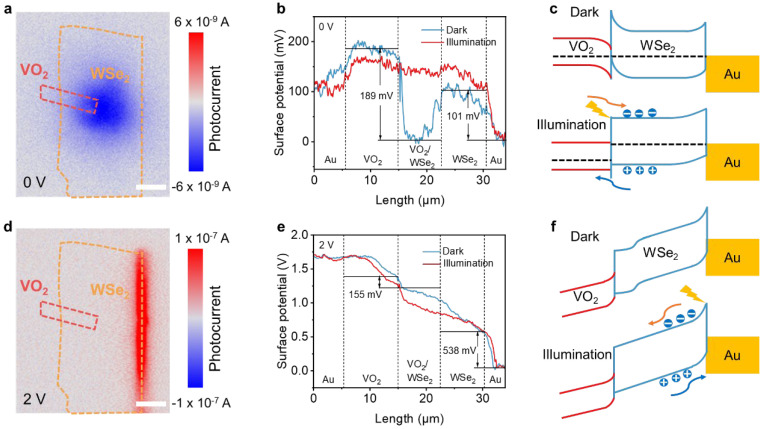
Retina-like working mechanism: (**a**,**d**) scanning photocurrent imaging of the device at bias voltages of 0 V and 2 V respectively. Scale bar, 9 μm. (**b**,**e**) Surface potential line profiles along the device under dark and illumination conditions at bias voltages of 0 V and 2 V respectively. (**c**,**f**) Energy band alignment in the device under dark and illumination conditions at bias voltages of 0 V and 2 V respectively.

To reveal the retina-like working mechanism of the photodetector, scanning photocurrent imaging is carried out in Figure 3a,d. It was found that at 0 V, the blue-contrast photoactive region locates around the VO_2_/WSe_2_ heterojunction in Figure 3a, with a negative photocurrent of around 6 nA. When the device operates at 2 V, the VO_2_/WSe_2_ heterojunction is deactivated, while the red-contrast region generates at the WSe_2_/Au interface in Figure 3d, showing a positive photocurrent of around 1000 nA. This confirms that switching the bias leads to a spatial shifting in the photoresponse area, corresponding to the design concept, as illustrated in Figure 1b, that VO_2_/WSe_2_ mimics the cone cell and WSe_2_/Au mimics the rod cell.

To further reveal the photoresponse-switching mechanism, surface potential profiles along the device were scanned using a Kelvin probe force microscope (KPFM) (Figure S6). As our KPFM works at the tip-biased mode, a lower surface potential implies a larger work function, and the real energy band bending is opposite to the change in surface potential [46,47,48]. As shown in Figure 3b, there is a 189 mV surface potential difference between the WSe_2_ and VO_2_, which is higher than the 101 mV of WSe_2_/Au. As a result, the WSe_2_/VO_2_ heterojunction plays the dominating role. Figure 3c schematically illustrates the energy band alignment according to the KPFM result, where electrons in the WSe_2_ flow into the VO_2_, leading to an electron depletion and upward band bending that forms a stronger built-in electric field. Under the illumination, excited electrons and holes are mainly separated by the built-in electric field to the WSe_2_ and VO_2_ sides, respectively. The interfacial energy band is then lifted and the surface potential recovers close to the flat band state as shown in red profile in Figure 3b, and the shifting of band alignment is shown in the illuminated diagram in Figure 3c.

Under 2 V bias, the external bias leads to a strong bending of the energy band, and thus a strong tilting of surface potential in Figure 3e. As the external field is opposite to the built-in electric field within the heterojunction, while coinciding with the electric field of the WSe_2_/Au Schottky junction, the potential difference in the heterojunction is then weakened from 189 mV to 155 mV, while the potential difference in the Schottky contact is enhanced from 101 mV to 538 mV. This change leads to a shifting in the carrier separation area of the WSe_2_/Au Schottky junction as shown in Figure 3f. It can be seen that under illumination, the voltage further drops on the Schottky junction, which makes it the major area for photoresponse. From the above band picture, it can be inferred that in dim light, the applied bias leads to more voltage drop on the Schottky junction which induces a more sensitive photoresponse than that at 0 V. However, the applied bias would lead to a more severe injection screening effect at high light intensities, causing the saturation of photoresponse as shown in Figure 2d. Figure 3e also shows that there is negligible voltage drop at the Au/VO_2_ contact, which means that it forms Ohmic contact and shows no contribution to the photoresponse.

### 3.4. Imaging Demonstration

**Figure 4:**
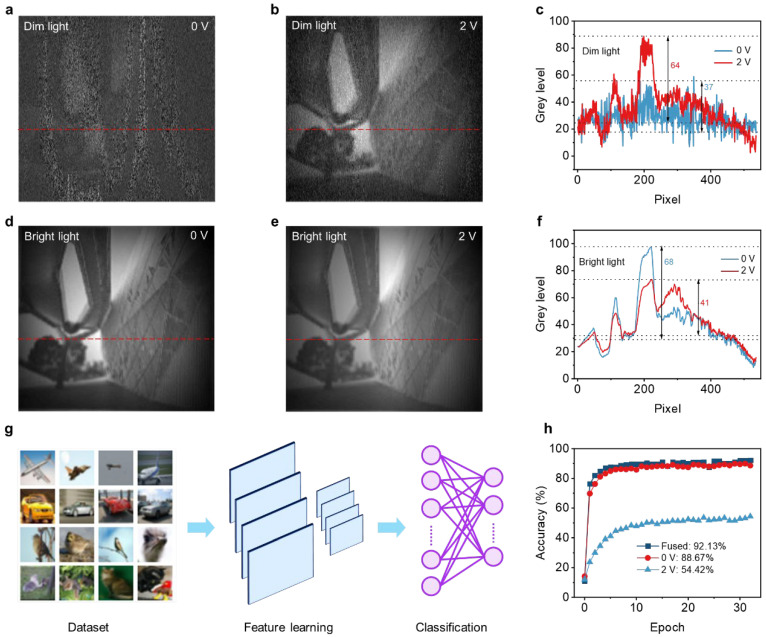
Demonstration of bionic visual imaging: (**a**,**b**) images of architectural film imaging in the 0 V mode (V_ds_ = 0 V) and the 2 V (V_ds_ = 2 V) respectively under dim light conditions. The red dotted line indicates the position where pixel values are extracted. (**c**) The pixel values of (**a**,**b**). (**d**,**e**) Images of architectural film imaging in 0 V mode and 2 V mode respectively under bright light conditions. The red dotted line indicates the position where pixel values are extracted. (**f**) The pixel values of (**d**,**e**). (**g**) Workflow diagram of convolutional neural network. (**h**) The recognition accuracy rates achieved by using different modes of the detector.

To demonstrate the ability to mimic the visual imaging function of the human eye, an imaging system in Figure S7a was set up for single-pixel imaging using the VO_2_/WSe_2_ heterojunction photodetector and a transparent architectural film was used as the imaging object in Figure S7b. An illumination of 27 μW cm^−2^ was used as the dim condition while an optical illumination of 2 mW cm^−2^ was used as the bright condition. The images formed in the 0 V mode and the 2 V mode under dim light conditions are shown in Figure 4a and Figure 4b respectively. The images captured in the 2 V mode have less noise and clearly display the image details, while in the 0 V mode, only the blurry outlines can be seen. The corresponding pixel differences are shown in Figure 4c. The pixel difference in the image captured in the 2 V mode is as high as 64, while that of the image captured in the 0 V mode is as low as 37. This is because the 2 V mode has a lower NEP compared to the 0 V mode. On the contrary, the images formed in the 0 V mode and the 2 V mode under the bright light condition are shown in Figure 4d and Figure 4e respectively. As shown in Figure 4f, the pixel difference in the image captured in the 0 V mode is as high as 68, while the pixel difference in the image captured in the 2 V mode is only 41. This indicates that the image captured in the 0 V mode has clearer details. This is because the 0 V mode still maintains a good linear response even under strong light conditions, while the 2 V mode exhibits a response characteristic of photoelectric current saturation under strong light. Therefore, the imaging results demonstrate that our device can effectively capture information in weak light in the 2 V mode, and can effectively capture information in strong light in the 0 V mode. In order to further demonstrate the characteristics of our device, we constructed a convolutional neural network as shown in Figure 4g. We used the CIFAR database as the dataset, which contains 60,000 32 × 32 color images divided into 10 categories, with 6000 images in each category. A total of 50,000 images were used for training and 10,000 images for testing. Using the VGG16 network of MATLAB (R2024b) to classify the CIFAR dataset, the specific process of image recognition is shown in Figure S8. The results are shown in Figure 4h. After 32 epochs, the recognition accuracy of the 0 V mode and 2 V mode was 88.67% and 54.42% respectively, while the image recognition rate when combining the two modes was 92.13%. This is because the dynamic range of the single 2 V mode (41.82 dB) and 0 V mode (122.03 dB) is small, which cannot accurately capture the image information. However, combining the two detection modes will achieve a larger dynamic range (146.66 dB). These results indicate that using the 0 V mode and 2 V mode of the VO_2_/WSe_2_ van der Waals heterojunction photodetector will effectively improve the ability to capture image information.

## 4. Conclusions

In conclusion, this study introduces a VO_2_/WSe_2_ van der Waals heterojunction photodetector where asymmetric contacts enable a dual-mode photoresponse for adaptive machine vision. At 0 V bias, this device operates in the cone mode and is capable of capturing optical information under strong light (5.4 μW). At 2 V bias, the device operates in the rod cell mode and is able to capture optical information under weak light (248.2 fW). A dynamic range of 146.66 dB was achieved through this dynamically switching detection mode, which is comparable to the level of the human eye. The fabrication of our VO_2_/WSe_2_ heterojunction by mechanical exfoliation and dry-transfer processes has demonstrated the feasibility of fabricating visual sensors. Further scaling-up requires the growth of large-scale 2D materials combined with a microfabrication process. This paves the way for the development of high-performance vision sensors.

## Figures and Tables

**Table 1:** Comparison of bio-inspired human-eye visual sensors.

Device Type	Working Mechanism	DR	Response Time	Reference	Published Time
Bilayer MoS_2_ (FET)	Defect adaptation	199 dB	5–120 s	[9]	2022
Cd (S, Se) (Photodiode)	Defect adaptation	193 dB	0.017/~18 s	[6]	2025
Si/Gr (FET)	Gate modulation	170 dB	5 ns/6 μs	[8]	2025
VO_2_/WSe_2_ (Photodiode)	Voltage modulation	146.66 dB	35.9/48.35 μs	Our work	

## Data Availability

The data that support the findings of this study are available from the corresponding author upon reasonable request.

## Supplementary Materials

The following supporting information can be downloaded at: https://www.mdpi.com/article/10.3390/nano16090498/s1, Figure S1 Growth of VO_2_: (a) VO_2_ micron band crystal optical image. (b) XRD spectrum of VO_2_ crystal. Figure S2 Device fabrication: schematic diagram of the VO_2_/WSe_2_ van der Waals heterojunction fabrication process. Figure S3 Material characterization: (a) AFM images of the VO_2_/WSe_2_ heterojunction. (b) PL at different positions of heterojunctions. Figure S4 Noise-current variation of VO_2_/WSe_2_ with frequency under different bias voltages. Figure S5. Photoresponse characterization: (a,b) Current-time curves of heterojunction under 2 V and 0 V bias voltages. (c,d) Current-optical power density curve of heterojunction under 2 V and 0 V bias voltages. Figure S6 Working mechanism characterization: (a) KPFM images of the device under dark and light conditions with a 0 V bias voltage. (b) KPFM images of the device under dark and light conditions with a 2 V bias voltage. Figure S7 Imaging application characterization: (a) Schematic representation of the imaging process. (b) The architectural images used as masks during the imaging process. Figure S8 Current-light power density curve obtained after logarithmically compressing the raw data of the device in Figure 2d. Table S1. DR Comparison of 2D Materials and Their Heterojunctions. Refs. [15,38,39,46,49,50,51,52,53,54,55,56,57,58,59,60] are cited in the supplementary materials.
