# Supplementary material for: Hybrid Junction-Enabled Biomimetic Human Eye Structure for Large Dynamic Range Vision Sensor
Source: Nanomaterials (Basel). 2026 Apr 22;16(9):498. doi: 10.3390/nano16090498 (PMC13164725; doi:10.3390/nano16090498)
Supplement: Supplementary file 1 [file nanomaterials-16-00498-s001.zip › nanomaterials-4236995-supplementary.pdf]

Supporting Information

Hybrid Junction-Enabled Biomimetic Human Eye Structure for Large Dynamic Range Vision Sensor

Daqi Chen [1], Yueheng Lu [1], Zhenye Zhan [1], Yuanfan Han [1], Zhendong Weng [2], Jian Chen [2], Qiulan Chen [3], Yang Zhou [1,]* and Weiguang Xie [1,4,]*

[1] Siyuan Laboratory, Guangdong Provincial Engineering Technology Research Center of Vacuum Coating Technologies and New Energy Materials, Department of Physics, Jinan University, Guangzhou 510632, China

[2] Instrumental Analysis & Research Center, Sun Yat-sen University, Guangzhou 510275, China

[3] Department of Medical Devices, Guangdong Food and Drug Vocational College, Guangzhou 510520, China

[4] Guangdong Provincial Key Laboratory of Optical Fiber Sensing and Communications, Jinan University, Guangzhou 510632, China

* Correspondence: yangzhou@email.jnu.edu.cn (Y.Z.); wgxie@email.jnu.edu.cn (W.X.)

Part I: Preparation of VO_2/WSe_2

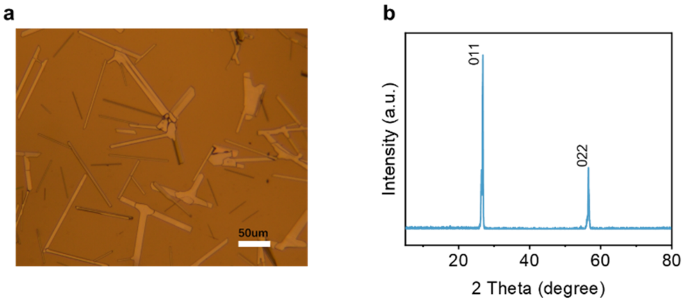

Figure S1 Growth of VO_2: a) VO_2 micron band crystal optical image. b) XRD spectrum of VO_2 crystal.

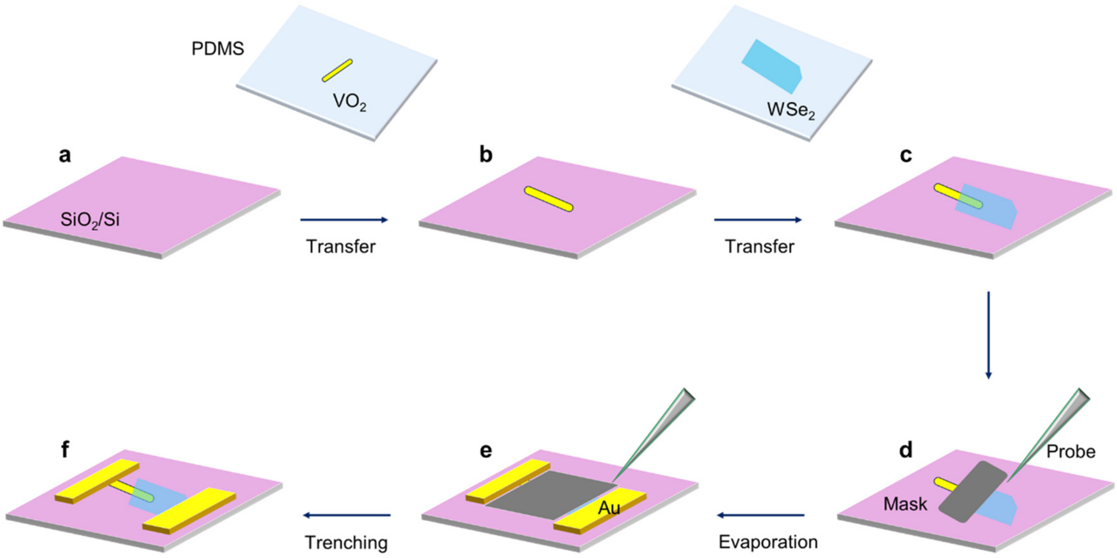

Figure S2 Device fabrication: schematic diagram of the VO_2/WSe_2 van der Waals heterojunction fabrication process.

Part II: Extended experimental data of sample characterization

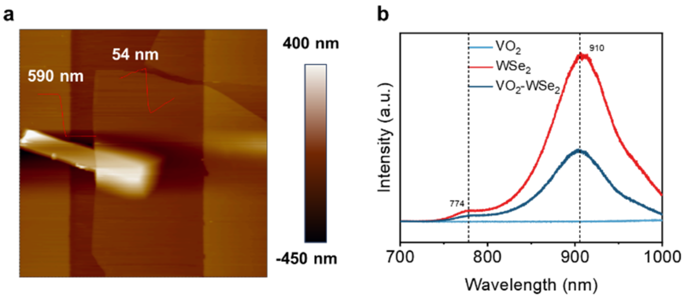

Figure S3 Material characterization: a) AFM images of the VO_2/WSe_2 heterojunction. b) PL at different positions of heterojunctions.

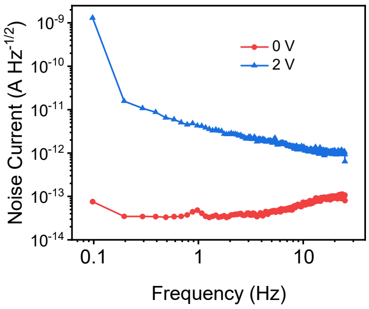

Figure S4 Noise-current variation of VO_2/WSe_2 with frequency under different bias voltages.

According to the calculation of equation (3), the NEP of the 0 V mode is approximately 128.43 fW $/\sqrt{Hz}$, and the NEP of the 2 V mode is approximately 19.31 fW $/\sqrt{Hz}$. It should be noted that equation (3) only considers the noise of the device in the dark state, excluding photon noise, source noise, background noise, etc., which results in its value being lower than the NEP obtained through direct measurement. Therefore, we take the actual test value shown in Figure 2b as the standard.

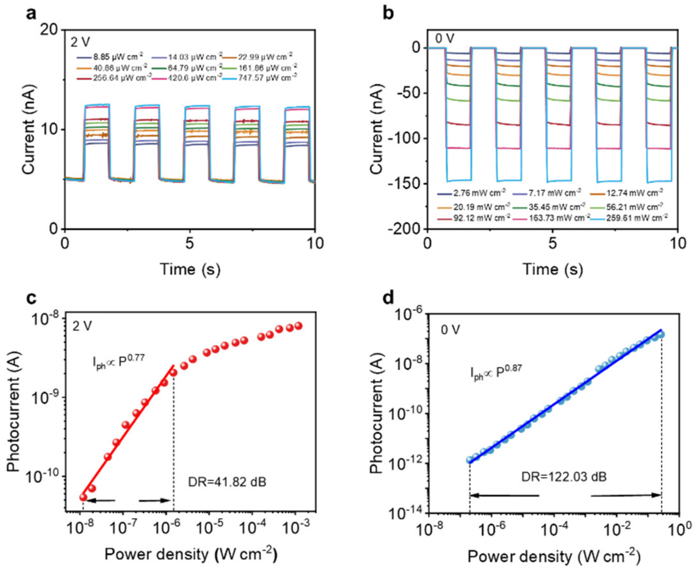

Figure S5 Photoresponse characterization： a-b) Current-time curves of heterojunction under 2 V and 0 V bias voltages. c-d) Current-optical power density curve of

heterojunction under 2 V and 0 V bias voltages.

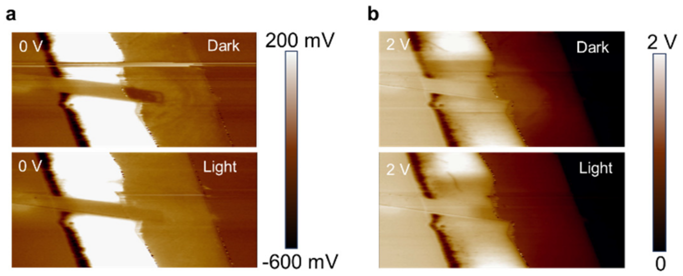

Figure S6 Working mechanism characterization: a) KPFM images of the device under dark and light conditions with a 0 V bias voltage. b) KPFM images of the device under dark and light conditions with a 2 V bias voltage.

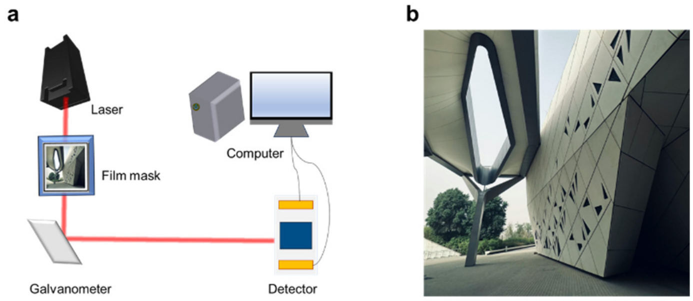

Figure S7 Imaging application characterization: a) Schematic representation of the imaging process. b) The architectural images used as masks during the imaging process.

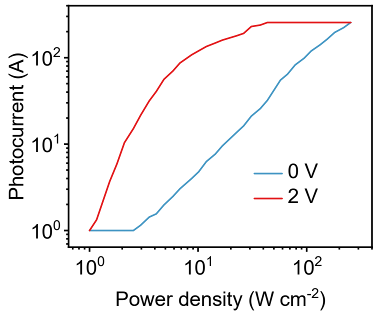

Figure S8 Current-light power density curve obtained after logarithmically compressing the raw data of the device in Figure 2d.

The raw device data shown in Figure 2d were transformed using a logarithmic function ($y=a\times\log(x)+b$) to compress the light power density into the range of 0–255. The

resulting curve is presented in Figure S8. Subsequently, the light power density values from this curve were assigned to image pixels, while the corresponding photocurrent values were used as pixel intensities. The CIFAR dataset was then mapped using this relationship to generate simulated imaging results. The CIFAR dataset was classified using the VGG16 network in MATLAB. The higher the image recognition accuracy, the more detailed the captured images, which indicates that the imaging quality of the detector is better.

Table S1. DR Comparison of 2D Materials and Their Heterojunctions

Device	DR	Responsivity	Response time	Reference
**Graphene**	70 dB	4.7×10^3 A/W	/	[1]
**Gr/$PdSe_2$/$MoSe_2$/Gr**	70.5 dB	651 mA/W	41.7/62.5 μs	[2]
**InSe/InGaSe**	74 dB	49 A/W	180/240 ms	[3]
**$PdSe_2$ P-I-N**	90 dB	1.1 A/W	3/6 μs	[4]
**MoS_2/Perovskite**	90 dB	2.5 A/W	109/150 μs	[5]
**α-In_2Se_3**	95.8 dB	6.37×10^4 A/W	20.5/24.9 μs	[6]
**$PtSe_2$/Si**	114 dB	520 mA/W	55.3/170.5 μs	[7]
**WSe_2/MoS_2**	123 dB	/	/	[8]
**WSe_2/$PdSe_2$**	142 dB	47.9 mA/W	8 ns	[9]
**$PtTe_2$/WS_2/Si**	144 dB	1.1 A/W	53/64 μs	[10]
**MoS_2/$BaTiO_3$**	152 dB	17402 A/W	20 ms	[11]
**VO_2/WSe_2**	146.66 dB	217 A/W	35.9/48.35 μs	Our work

Part III: Calculation of linear dynamic range (DR), responsivity (R), noise equivalent power (NEP) and specific detectivity (D^*)

The linear dynamic range (DR) is defined as:

$$DR = 20\log\frac{P_{max}}{P_{min}} \quad (1)$$

P_{max} represents the optical power at which the photocurrent deviates from the linear behavior and tends to saturation, while P_{min} represents the noise equivalent power (NEP).[12] The responsivity (R) is defined as:

$$\mathrm{R} = \frac{I_P}{\mathrm{P}} \quad (2)$$

I_P represents photocurrent and P represents effective laser power.[13] Noise equivalent power (NEP) can be estimated using the following formula:

$$NEP = \frac{i_n}{\mathrm{R}} \quad (3)$$

i_n represents the root mean square of the noise current, and R represents the response responsivity.[14] The specific detectivity (D^*) is defined as:

$$D^* = \frac{\sqrt{S \cdot \nabla f}}{i_n} \quad (4)$$

S represents the effective illuminated area (here we use the area within the device channel), ∇f is the electrical bandwidth.[15,16]

References

1. Nashashibi, S.; Koepfli, S.M.; Schwanninger, R.; Baumann, M.; Doderer, M.; Bisang, D.; Fedoryshyn, Y.; Leuthold, J. Engineering Graphene Phototransistors for High Dynamic Range Applications. *ACS Nano* **2024**, *18*, 12760–12770, doi:10.1021/acsnano.3c11856.
2. Zhong, J.; Wu, B.; Madoune, Y.; Wang, Y.; Liu, Z.; Liu, Y. $PdSe_2$/$MoSe_2$ Vertical Heterojunction for Self-Powered Photodetector with High Performance. *Nano Res.* **2022**, *15*, 2489–2496, doi:10.1007/s12274-021-3745-9.
3. Yu, M.; Hu, Y.; Gao, F.; Dai, M.; Wang, L.; Hu, P.; Feng, W. High-Performance Devices Based on InSe–$In_{1-x}Ga_xSe$ Van Der Waals Heterojunctions. *ACS Appl. Mater. Interfaces* **2020**, *12*, 24978–24983, doi:10.1021/acsami.0c03206.
4. Jiang, J.; Xu, W.; Guo, F.; Yang, S.; Ge, W.; Shen, B.; Tang, N. Polarization-Resolved Near-Infrared PdSe2 p-i-n Homojunction Photodetector. *Nano Letters* **2023**, *23*, 9522–9528, doi:10.1021/acs.nanolett.3c03086.
5. Lu, Y.; Zhan, Z.; Tan, J.; Lai, H.; Liu, P.; Zhou, Y.; Xie, W. High-performance MoS_2 Homojunction Photodiode Enabled by Facile Van Der Waals Contacts with 2D Perovskite. *Laser & Photonics Reviews* **2024**, *18*, 2300941, doi:10.1002/lpor.202300941.
6. Lu, Y.; Zhan, Z.; Tan, J.; Weng, Z.; Chen, J.; Zhou, Y.; Xie, W. Defect-Mediated Exciton Dissociation in α-In_2Se_3 for Photodetection with High-Efficiency Carrier Collection. *ACS Photonics*, 2025, 12(11): 6438-6447.
7. Xie, C.; Zeng, L.; Zhang, Z.; Tsang, Y.-H.; Luo, L.; Lee, J.-H. High-Performance Broadband Heterojunction Photodetectors Based on Multilayered $PtSe_2$ Directly Grown on a Si Substrate. *Nanoscale* **2018**, *10*, 15285–15293, doi:10.1039/c8nr04004d.

8. Lee, H.S.; Ahn, J.; Shim, W.; Im, S.; Hwang, D.K. 2D WSe_2/MoS_2 van Der Waals Heterojunction Photodiode for Visible-near Infrared Broadband Detection. *Applied Physics Letters* **2018**, *113*, doi:10.1063/1.5042440.
9. Wen, S.; Zhou, S.; Gong, Y.; Zhang, R.; Jia, X.; Kong, L.; Fan, H.; Yin, Y.; Lan, C.; Li, C.; et al. An Ultra-Fast WSe_2 Homojunction Photodiode with a Large Linear Dynamic Range towards in-Sensor Image Processing. *Nanoscale Horizons* **2025**, *10*, 885–895, doi:10.1039/D4NH00656A.
10. Zhou, S.; Wen, S.; Fan, H.; Wei, Y.; Yin, Y.; Lan, C.; Li, C.; Liu, Y. $PtTe_2/WS_2$/Pyramidal-Si van Der Waals Heterojunction with Semiconformal Interfaces toward High-Performance Photodetectors. *ACS Photonics* **2024**, *11*, 1810–1820, doi:10.1021/acsphotonics.4c00331.
11. Guan, H.; Liu, F.; He, Z.; Xie, H.; Xie, M.; Fang, Z.; Yang, M.; Chen, B.; Liang, X.; Li, F.; et al. Efficient Electrical Extraction of Nonlinear Response and Large Linear Dynamic Range Implementation in $MoS_2/BaTiO_3$ Hetero-Integrated Photodetector. *Laser & Photonics Reviews* **2024**, *18*, 2400445, doi:10.1002/lpor.202400445.
12. Wang, Q.; Zhang, Y.; Wei, Z. Recent Progress on Organic NEAR-INFRARED Photodetectors: Mechanism, Devices, and Applications†. *Chin. J. Chem.* **2023**, *41*, 958–978, doi:10.1002/cjoc.202200686.
13. Lu, Y.; Sun, X.; Zhou, H.; Lai, H.; Liu, R.; Liu, P.; Zhou, Y.; Xie, W. A High-Performance and Broadband Two-Dimensional Perovskite-Based Photodetector via van Der Waals Integration. *Applied Physics Letters* **2022**, *121*, 161104, doi:10.1063/5.0116505.
14. Liu, X.; Deng, C.; Wei, H.; Fang, M.; Yan, B.; Zhu, T.; Luo, S.; Peng, G.; Cai, W.; Long, M.; et al. High-Sensitive Uncooled Mid-Wave Infrared Photodetector Based on $PtSe_2/MoTe_2$ Heterojunction with Fast Speed. *Advanced Functional Materials* **2025**, *35*, 2423102, doi:10.1002/adfm.202423102.
15. Wang, F.; Zhang, T.; Xie, R.; Wang, Z.; Hu, W. How to Characterize Figures of Merit of Two-Dimensional Photodetectors. *Nat Commun* **2023**, *14*, 2224, doi:10.1038/s41467-023-37635-1.
16. Teng, F.; Hu, K.; Ouyang, W.; Fang, X. Photoelectric Detectors Based on Inorganic p-Type Semiconductor Materials. *Advanced Materials* **2018**, *30*, 1706262, doi:10.1002/adma.201706262.
